# *Mycobacterium bovis* Infection in Humans and Cats in Same Household, Texas, USA, 2012

**DOI:** 10.3201/eid2103.140715

**Published:** 2015-03

**Authors:** Kira E.F. Ramdas, Konstantin P. Lyashchenko, Rena Greenwald, Suelee Robbe-Austerman, Cynthia McManis, W. Ray Waters

**Affiliations:** Just Cats Veterinary Services, The Woodlands, Texas, USA (K.E.F. Ramdas, C. McManis);; Chembio Diagnostic Systems, Inc., Medford, New York, USA (K.P. Lyashchenko, R. Greenwald);; National Veterinary Services Laboratories, Ames, Iowa, USA (S. Robbe-Austerman);; National Animal Disease Center, Ames (W.R. Waters)

**Keywords:** Mycobacterium bovis, tuberculosis, zoonosis, serology, whole genome sequencing, cats, Tuberculosis and other mycobacteria

## Abstract

*Mycobacterium bovis* infection of cats is exceedingly rare in regions where bovine tuberculosis is not endemic. We describe the diagnosis and clinical management of pulmonary *M. bovis* infection in 2 indoor-housed cats and their association with at least 1 *M. bovis*–infected human in Texas, USA, in September 2012.

Tuberculosis in humans and animals results from infection by bacilli within the *Mycobacterium tuberculosis* complex (*1*). Despite ≈99.95% genome sequence identity, *M. bovis* and *M. tuberculosis* exhibit distinct differences in host adaptation and susceptibility (*2*). *M. bovis* is the primary causative agent of bovine tuberculosis and infects a wider range of hosts than *M. tuberculosis*. In domestic cats, tuberculosis is caused primarily by infection with *M. bovis* or *M. microti* (*3**–**5*); *M. tuberculosis* infection is less common (*6*).

Before implementation of bovine tuberculosis control programs and wide-scale pasteurization of milk, alimentary tract disease was the most common form of tuberculosis in cats (*7*); today, lymphadenopathy and cutaneous forms are more common (*4*). Diagnosis is based on clinical examination, imaging, biopsy with histopathologic examination, culture of aspirates or tissues, and specific immune-based blood assays (*4*,*8*,*9*). Intradermal skin tests are generally unreliable for diagnosing tuberculosis in cats (*10*). Client history is critical for determining the possibility for exposure of the cat to the pathogen, and zoonotic aspects should be considered (*11*). We describe the diagnosis and clinical management of pulmonary *M. bovis* infection in 2 indoor-housed cats and their association with at least 1 *M. bovis*–infected human in Texas, USA.

## Case Report

In September 2012, a 5-year-old female domestic cat (cat Y) was seen by a veterinarian for dyspnea, tachypnea, hyporexia, and lethargy. She lived indoors with 4 other cats and their female owner. The vague history provided by cat Y’s owner indicated that, ≈11 months earlier, her husband had died of tuberculosis only 6 weeks after diagnosis and initiation of directly observed antimycobacterial therapy. At the time the husband’s tuberculosis was diagnosed, the woman was Mantoux-test negative; ≈2 months after his death, she converted to skin-test positive but had normal findings on thoracic radiographs. She was subsequently treated with antimycobacterial drugs. The woman also reported that, in June 2012, another cat in the household was euthanized after clinical signs developed that were similar to those of cat Y; no necropsy was performed. Additional pertinent history included relocation of the deceased husband from Mexico to Texas 15 years earlier, frequent contact with recent immigrants from Central America and Mexico, and consumption of unpasteurized Mexican cheeses.

**Figure 1:**
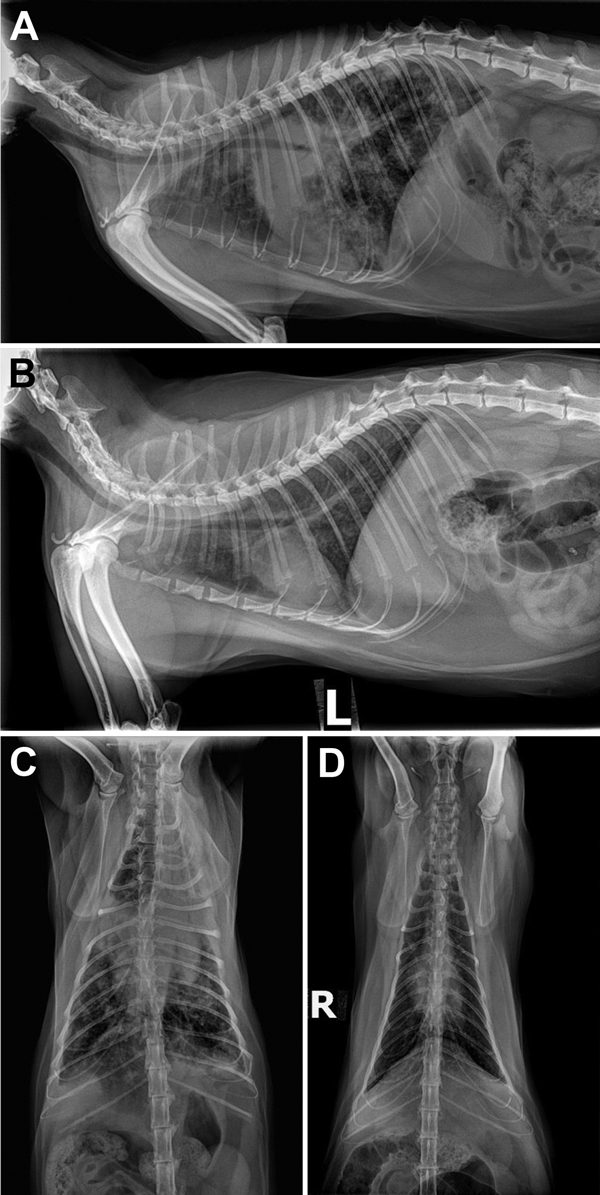
Radiograph images of cat Y showing pulmonary lesions before and after antimycobacterial treatment for *Mycobacterium bovis* infection, Texas, USA, 2012. A) Pretreatment, right lateral thoracic radiograph showing severe coalescing interstitial to alveolar pulmonary infiltrates before treatment. B) Posttreatment, left lateral thoracic radiograph after 2 months of marbofloxacin, rifampin, and a macrolide for 2 months in cat Y and then another 3.5 months of rifampin and marbofloxacin alone. C) Pretreatment, ventrodorsal view showing severe bronchointerstitial disease with poorly defined nodules or complete consolidation in the perihilar region, right middle lung lobe, and cranial segment of the left cranial lung lobe. D) Posttreatment, ventrodorsal thoracic radiographs after 2 months of triple antimycobacterial therapy and then another 3.5 months of rifampin and marbofloxacin alone. Considerable improvement occurred after therapy: the perihilar region cleared but a heavy interstitial marking throughout the lungs remained, most suggestive of fibrosis from scarring or, less likely, from smaller active granulomata.

On examination, cat Y had a productive cough with blood-tinged sputum, increased respiratory effort, and dorsal muscle wasting. A complete blood cell count revealed an elevated total leukocyte count, elevated neutrophil count, and mild anemia, consistent with chronic inflammatory disease. Abdominal and thoracic radiographs (Figure 1) showed severe bronchointerstitial consolidating lung disease with poorly defined nodules, tracheobronchial lymphadenopathy, and mild hepatosplenomegaly. Lung aspiration and cultures were performed under general anesthesia. Bloody sputum was noted in the endotracheal tube. Aerobic and anaerobic cultures and acid-fast bacilli (AFB) stains of aspirated samples were negative, and cytologically the samples were identified as mixed-cell inflammation. Given the history and clinical suspicion of tuberculosis, rifampin, marbofloxacin, and clarithromycin were prescribed, along with isolation of cat Y in a separate room in the household. Cat Y responded favorably to empirical treatment with antimycobacterial drugs (Figure 1).

**Figure 2:**
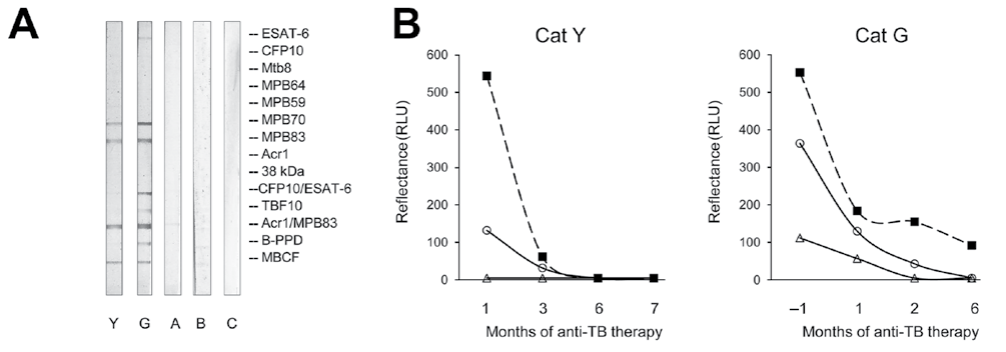
Antibody test results for cats tested for *Mycobacterium bovis* infection, Texas, USA, 2012. A) Antibody reactivity in infected (Y, G) and presumed noninfected (A–C) cats. Images show MAPIA strips processed with serum samples as described previously (*13**,**14*). Visible bands reflect the presence of IgG to *Mycobacterium bovis* antigens indicated in the right margin. B) Antibody levels in infected cats Y and G during antimycobacterial therapy. Intensity of test bands in TB STAT-PAK (solid squares) and DPP VetTB assay (circles, MPB83 antigen; triangles, ESAT-6/CFP10 antigen) was measured by optical reader. The TB STAT-PAK assay can detect both IgM and IgG; DPP VetTB detects IgG only (all assays from Chembio Diagnostic Systems, Inc., Medford, NY, USA).

One month after cat Y was initially seen for care, all 5 cats in the household (cat Y, cats A–C, and cat G) were brought for screening thoracic radiographs and serology. Cats A–C and G exhibited no respiratory signs and appeared clinically healthy. Lung radiographs of cat G showed perihilar nodular changes and consolidation consistent with granulomas. The 5 cats were tested by 3 antibody-detection assays, MAPIA, TB STAT-PAK, and DPP VetTB (Chembio Diagnostic Systems, Inc., Medford, NY, USA), known to detect specific antibody in feline *M. bovis* or *M. microti* infection (*8*,*12*). Cats Y and G were seropositive by all 3 immunoassays upon initial testing; the other cats (cats A, B, and C) were seronegative (Figure 2). Serum from cats Y and G reacted with MPB70 and MPB83 antigens. Serum from cat G also reacted to ESAT-6 protein and its fusion with CFP10 (Figure 2, panel A). The feline antibody profiles were consistent with the known serologic immunodominance of these antigens in *M. bovis* infection reported for other host species (*13*). As with elephants (*14*), serum antibody levels in cats Y and G gradually declined after 1–3 months of antimycobacterial treatment (Figure 2, panel B). Cats A–C remained antibody negative in all 3 immunoassays during the same period (data not shown).

Given the clinical and serologic findings, the treating veterinarian contacted the appropriate public health authorities, and the owner was instructed to continue isolation of cats Y and G because of the clinical, serologic, and radiographic evidence suggestive of tuberculosis. Efforts to obtain her husband’s medical records to aid in the cats’ diagnosis had been refused until positive serologic test results for cats Y and G were produced (Figure 2), after which permissions from surviving family members were obtained. A local public health clinic, where the deceased husband had received diagnosis and treatment for pulmonary tuberculosis, provided results of sputum culture. These results showed that *M. bovis* had been isolated from his sputum; however, a previous investigation had not elucidated a definitive source of infection. After the cats’ veterinarian received this new information, bronchoalveolar lavages and gastric washes were performed on cats Y and G. AFB were detected in cat G’s gastric wash, and *M. bovis* was isolated by culture conducted at the National Veterinary Services Laboratories (Ames, IA, USA). No mycobacterial growth was obtained from cat Y, presumably because of the 7 weeks of antimycobacterial therapy before sampling. Antimycobacterial therapy (rifampin, marbofloxacin, and clarithromycin) was initiated for asymptomatic cat G. Whole-genome sequencing performed at the National Veterinary Services Laboratories revealed that the isolate from the husband and cat G were closely related but not identical; there were 8 single nucleotide polymorphisms (SNPs) between the 2 isolates (Table). As of April 8, 2014, all 5 cats and the owner remained clinically well.

**Table:** Single nucleotide polymorphism differences between the isolates from a cat and a human with *Mycobacterium bovis*, Texas, USA, 2012*

Genome position aligned to the reference *M. bovis* AF2122/97	1859572	3639316	586911	2567	2847080	3394685	3603337	80817
Reference call	**C**	**C**	**G**	**C**	**G**	**T**	**T**	**T**
12–9271_TX_Cat_Domestic	**T**	**A**	**A**	C	G	T	T	T
13–0751_TX_Human	C	C	G	**T**	**C**	**C**	**G**	**C**

*The sequences were aligned against the reference genome, *M. bovis* AF2122/97 (reference call). Boldface highlights the different amino acid at each single nucleotide polymorphism between the 2 genotypes.

## Conclusions

Our findings highlight the possibility of *M. bovis* infection in an unlikely geographic locale (Houston, Texas) to which bovine tuberculosis is not endemic and an unlikely population (cats and humans living in the same household). The owners and their cats had no known contact with cattle or wildlife; however, the deceased husband had relocated to Texas from a country where bovine tuberculosis is considered endemic. The family also had frequent visitors and consumed unpasteurized dairy products from bovine tuberculosis–endemic countries. The cat that had died with signs consistent with pulmonary tuberculosis spent extended close contact with the owner, who died of pulmonary *M. bovis* infection. All cats in the household had close and frequent contact with both owners. Whole-genome sequencing indicated that the cat and human *M. bovis* isolates, although closely related, were either 3 or 5 SNPs from sharing a common ancestor. Previous research suggests variable SNP accumulations (0–2) per transmission event, which implies that direct transmission between the owner and cat G is unlikely (*15*). However, whether the isolates recovered reflect all the genotypes within the individual is unknown. A clinically relevant finding was the use of serologic testing to justify additional procedures necessary for isolating *M. bovis* and assisting with treatment monitoring. These findings highlight the complexity of diagnosing *M. bovis* infection in an unanticipated host and setting.
